# Depressive symptoms and alcohol correlates among Brazilians aged 14 years and older: a cross-sectional study

**DOI:** 10.1186/1747-597X-9-29

**Published:** 2014-07-15

**Authors:** Cassiano L S Coelho, Ronaldo R Laranjeira, Jair L F Santos, Ilana Pinsky, Marcos Zaleski, Raul Caetano, José Alexandre S Crippa

**Affiliations:** 1 Department of Psychiatry, Universidade Federal de São Paulo (UNIFESP), R. Felício Antônio Siqueira, 2068, Higienópolis, CEP 15085-420, São José do Rio Preto, SP, Brazil; 2 Department of Social Medicine of the Ribeirão Preto Medical School, Universidade de São Paulo (FMRP-USP), Av. Dos Bandeirantes, 3900, Monte Alegre, CEP 14049-900 Ribeirão Preto, SP, Brazil; 3 Departamento de Clínica Médica, Universidade Federal de Santa Catarina (UFSC), Centro de Ciências da Saúde, Campus Universitário, Trindade, CEP 88040-900 Florianópolis, SC, Brazil; 4 Dallas Regional Campus, University of Texas, School of Public Health, 5323 Harry Hines Blvd., TX 75390 Dallas, Texas, USA; 5 Department of Neuroscience and Behavior of the Ribeirão Preto Medical School, Universidade de São Paulo (FMRP-USP), Av. Bandeirantes, 3900, Monte Alegre, CEP 14048-900 Ribeirão Preto, SP, Brazil; 6 INCT Translational Medicine, Sao Paulo, Brazil

**Keywords:** Depressive symptoms, Alcohol dependence, Epidemiology, Survey, Comorbidity

## Abstract

**Background:**

The associations between depressive symptoms and alcohol-related disorders, drinking patterns and other characteristics of alcohol use are important public health issues worldwide. This study aims to study these associations in an upper middle-income country, Brazil, and search for related socio-demographic correlations in men and women.

**Methods:**

A cross-sectional study was conducted between November 2005 and April 2006. The sample of 3,007 participants, selected using a multistage probabilistic sampling method, represents the Brazilian population aged 14 and older. Depressive symptoms were assessed using the Center for Epidemiologic Studies Depression Scale and alcohol dependence was assessed using the Composite International Diagnostic Interview. Associations assessed using bi-variate analysis were tested using Rao-Scott measures. Gender specific multinomial logistic regression models were developed.

**Results:**

Among the participants with alcohol dependence, 46% had depressive symptoms (17.2% mild/moderate and 28.8% major/severe; *p* < 0.01); 35.8% (*p* = 0.08) of those with alcohol abuse and 23.9% (*p* < 0.01) of those with a binge-drinking pattern also had depressive symptoms. Alcohol abstainers and infrequent drinkers had the highest prevalence of major/severe depressive symptoms, whereas frequent heavy drinkers had the lowest prevalence of major/severe depressive symptoms. In women, alcohol dependence and the presence of one or more problems related to alcohol consumption were associated with higher risks of major/severe depressive symptoms. Among men, alcohol dependence and being ≥45 years old were associated with higher risks of major/severe depressive symptoms.

**Conclusions:**

In Brazil, the prevalence of depressive symptoms is strongly related to alcohol dependence; the strongest association was between major/severe depressive symptoms and alcohol dependence in women. This survey supports the possible association of biopsychosocial distress, alcohol consumption and the prevalence of depressive symptoms in Brazil. Investing in education, social programs, and care for those with alcohol dependence and major/severe depressive symptoms, especially for such women, and the development of alcohol prevention policies may be components of a strategic plan to reduce the prevalence of depression and alcohol problems in Brazil. Such a plan may also promote the socio-economic development of Brazil and other middle-income countries.

## Introduction

According to the World Health Organization (WHO), depression is estimated to be the leading cause of years lost to disability (YLD) in males and females. Furthermore, alcohol-related disorders are the second leading cause of YLD in males [1]. Most clinical and epidemiological studies have found that depression is two to three times more frequent among women than among men [2,3] and alcohol-related disorders are significantly more prevalent among men [4]. Both represent an enormous burden to individuals and society [5].

Over the last few decades, many international epidemiologic surveys have consistently observed a positive association between depression and alcohol-related disorders [6,4]. It has been proposed that this relationship can be either artifactual, causal, or due to shared etiology.

The artifactual theory explains the relationship between alcohol and depressive symptoms as an artifact of study methodologies [7].

Causal theories suggest that heavy drinking or alcohol use/abuse may cause depression, in the short-term, due to the pharmacological effects of alcohol [8]. In the long term, neurobiological studies have demonstrated that depression could result from hippocampal atrophy [9]. Other studies claim that alcohol-related disorders lead to an increased risk of major depression [10], as opposed to a self-medication model, in which major depression leads to increased risk of alcohol abuse or dependence [11].

The shared etiology theory proposes that chronic drinking and related symptoms may indirectly promote depression as well, such as by contributing to stressful life circumstances (e.g., problems related to alcohol consumption) that in turn are known to promote depression [12]. Biopsychosocial distress could lead to both problems [13].

Although studies have investigated the associations between depression and alcohol use, no single definitive causal or shared etiological risk factor has been found to underlie both disorders [14].

Epidemiologic studies have examined the association between depression and the characteristics of alcohol consumption, such as frequency, volume, and binge drinking, within gender and other sociodemographic strata. These studies found a non-linear association evident from classical J- or U-shaped curves (this type of curve reflects higher frequencies of depressive symptoms among abstainers and heavy drinkers and the lowest frequencies among light or moderate drinkers). The study results vary, probably because a different approaches were used to measure both depressive symptoms and alcohol consumption [15-17]. Some studies have even suggested that, among those with alcohol use disorders, gender differences in rates of depression may merely reflect the higher rate of depression observed among women in the general population rather than a stronger link between depression and measures of substance use and impairment [18,19]. Therefore, measurement methods and gender are key issues to consider when interpreting findings about the relationship between alcohol use and depression [16]. It is widely established that among alcohol dependent individuals major depression is more prevalent in women than in men [20,21]. A better understanding of the nature of these associations in men and women is very important because both conditions are highly prevalent and greatly impact public health [4].

High quality, country-specific data on the prevalence of different types of mental disorders are lacking for countries, such as Brazil [22], that are defined as upper middle-income economy countries according to the World Bank [23]. Consequently, findings from wealthier countries are often extrapolated to other parts of the world. Such generalizations may be misleading, particularly if the determinants of depression vary in different regions of the world [24]. Brazil has only recently begun comprehensive population-based studies of the association between depressive symptoms and different patterns of alcohol consumption and other correlates of alcohol use. A recent study conducted in an urban sample of the metropolitan region of São Paulo, in the State of São Paulo, southeast Brazil, reported that the association between depression and alcohol consumption differed between genders. In men, depression was associated with their alcohol-drinking pattern, mostly binge drinking, and to the problems caused by alcohol use. In women, depression was associated with residing with alcoholic spouses [25]. A cross-sectional study of high school students, also living São Paulo, detected a positive association between depressive symptoms, female gender and alcohol consumption [26]. Finally, another cross-sectional study of a representative sample of early adolescents living in an urban area of the city of Pelotas, State of Rio Grande do Sul, south Brazil, reported high depressive symptomatology associated with abuse of alcohol in the previous month [27].

The association between depressive symptoms and alcohol use disorders or other alcohol correlates has not been investigated in the entire Brazilian population. Specifically, such studies are lacking from the north and northeast regions of the country. Using data collected in the first Brazilian National Alcohol Survey (BNAS) [28], we aimed to estimate the prevalence of depressive symptoms in relation to alcohol-related disorders, drinking patterns and other alcohol use indicators in a representative sample of the Brazilian population aged 14 years and older and to examine the correlations between these conditions and socio-demographic characteristics in men and women. Our goal is to expand the knowledge regarding these associations in an upper middle-income country, such as Brazil.

## Methods

### Sample

The study sample was obtained in the first BNAS [28] conducted by the Federal University of São Paulo (UNIFESP) Unit of Studies on Alcohol and Other Drugs (UNIAD), São Paulo, Brazil. Between November 2005 and April 2006, multistage cluster sampling was used to select 3,007 individuals aged 14 years and older who represented a sample of the entire Brazilian population, excluding native Brazilians who live in Indian reservations and populations who live in communities such as prisons. The survey covered 143 Brazilian cities and within them, 325 census sectors, including those situated in rural areas. One hour face-to-face interviews were conducted at the respondents’ home by trained interviewers. The response rate of the survey was 66.4%. All respondents provided a signed informed consent to participate. They were told that this was a national study and that their participation was important to guide future government public policies.

The data collection methods and the socio-demographic characteristics of the sample have been described in more detail elsewhere [28]. A summary of the demographic characteristics is shown in Table 1.

**Table 1:** Characteristics of the study sample, by gender

**Demographics and alcohol correlates**	**Male**		**Female**		**Total**		***P-value***
	**(%)**	**(n)**	**(%)**	**(n)**	**(%)**	**(n)**	
**Age group**							
14 – 17	10.9	335	9.8	326	10.3	661	*0.65*
18 – 24	17.9	159	16.9	209	17.4	368	
25 – 34	21.5	238	20.8	350	21.1	588	
35 – 44	19.3	192	19.2	296	19.2	488	
45 – 59	18.8	200	19.6	301	19.2	501	
≥ 60	11.6	161	13.8	240	12.7	401	
**Marital status**							
Single	36.4	560	31	596	33.6	1156	< 0.01
Married/Partner	58.2	631	52.6	814	55.3	1445	
Divorced/Separeted/Widowed	5.4	94	16.4	312	11.1	406	
**Schooling**							
Illiterate/Basic	34.5	430	34.4	587	34.4	1017	*0.62*
Incomplete or complete elementary school	29.3	402	26.9	470	28	872	
Incomplete or complete high school	27	391	29.1	559	28.1	950	
Incomplete or complete higher education	9.2	62	9.4	106	9.4	168	
**Family income**							
≤ US $208	34.5	495	39.2	740	36.9	1235	< 0.01
US $209 – US $347	18.2	259	19.2	325	18.7	584	
US $348 – US $555	20.9	226	16.6	257	18.7	483	
US $556 – US $1,157	14.1	143	11.2	160	12.6	303	
> US $1,157	6.1	55	4.8	65	5.4	120	
Not informed	6.2	107	9	175	7.7	282	
**Region**							
North	9.3	94	8	114	8.6	208	*0.83*
Mid-west	6.3	96	7.1	140	6.7	236	
Northeast	28.7	378	29.1	506	28.9	884	
Southeast	43.9	545	44.8	730	44.4	1275	
South	11.8	172	11.0	232	11.4	404	
**Sector**							
Urban	85.3	1074	84.1	1451	84.7	2525	*0.38*
Rural	14.7	211	15.9	271	15.3	482	
**Age at first use**							
No drinking	32	478	54.7	939	43.7	1417	< 0.01
Up to 14 years	13	194	5.3	127	9.1	321	
15 - 17 years	27.7	298	12.8	221	20	519	
18-24 years	23.3	246	21.1	308	22.2	554	
≥ 25 years	4	51	6.1	96	5.1	147	
**Problems related to alcohol consumption**							
0	62.6	871	88.8	1516	76.2	2387	< 0.01
1	7.4	85	4.6	71	5.9	156	
2	5.7	64	2	40	3.8	104	
3	5.1	54	1.1	23	3	77	
≥ 4	19.2	211	3.6	72	11.1	283	
**Volume-Variability Index**							
Frequent heavy drinker	13.5	146	2.9	55	8	201	< 0.01
Frequent drinker	20.5	229	8.3	140	14.2	369	
Less frequent drinker	15.2	176	12.9	217	14	393	
Infrequent drinker	12.5	166	15.4	250	14	416	
Abstainer	38.3	568	60.4	1060	49.7	1628	
**Binge drinking**							
Abstainer	49.7	567	60.4	1060	49.7	1627	< 0.01
No	24.2	294	22.9	373	23.5	667	
Yes	37.6	424	16.7	289	26.8	713	
**Abuse**							
No	85.3	1119	97.7	1671	91.7	2790	< 0.01
Yes	14.7	166	2.3	51	8.3	217	
**Dependence**							
No	87.1	1139	97	1660	92.2	2799	< 0.01
Yes	12.9	146	3	62	7.8	208	

The survey employed a version of the questionnaire used in the Hispanic Americans Baseline Alcohol Survey (HABLAS) [29], which examined various aspects of drinking and alcohol-related problems among these four Hispanic national groups in the United States: Puerto Ricans, Cuban Americans, Mexican Americans, and South/Central Americans. The questionnaire was translated by the survey’s coordinators and underwent a process of adaptation to the socio-cultural reality of the Brazilian population. The full Portuguese version of the questionnaire is available at http://www.uniad.org.br[30].

The study was approved by the Ethics Committee of the UNIFESP (process number CEP 1672/04).

### Variables

#### Alcohol use indicators

a) Age at first use

Age at first use was addressed through the question, “How old were you when you started to consume alcoholic beverages? Do not take into account those occasions when you had only one or two sips”.

b) Problems related to alcohol consumption

There were 28 questions about possible problems originating from consumption in the prior 12 months that were classified into six areas: social, working, family, legal, physical, and those related to violence (Possible answers: 0, 1, 2, 3 or more than 4 problems in any of these six areas). Examples of how these areas were investigated: Social, “I had a heated discussion while drinking” or “My alcohol consumption contributed to my being involved in an accident in which someone else was hurt or something, such as a car, was damaged”; Working, “Drinking may have affected my chances of being promoted, being given a salary raise, or a job benefit” or “I almost lost or I have lost a job because of drinking”; Family, “I realized that drinking has caused me problems with my children” or “My husband/wife has threatened to leave me because of my drinking”; Legal, “Drinking has caused me legal problems” or “A police officer warned me about my drinking”; Physical, “My hands were shaking strongly the morning after I had drunk” or “I felt that drinking was becoming a serious threat to my physical health”; and Violence, “I have hit or attacked someone while drinking” or “I started a fight with someone outside of my family when I was drinking”.

c) Volume-Variability Index

A modified version of the Cahalan and Cisin [31] Volume-Variability (V-V) Index was used to classify respondents’ patterns of alcohol consumption. Respondents were asked about their consumption of wine, beer, liquor, and “alcopops” in the 12 months prior to the interview. The respondents’ frequency of drinking was coded into 11 categories, ranging from “never” to “three or more times a day”. The quantity of consumption was assessed by asking for the frequency of drinking 1 to 12 drinks of wine, beer or liquor. These quantities were asked using these ranges: 1, 2, 3, 4, 5 to 7, 8 to 11, 12 or more. The unit of measure used in this study was “one drink”, corresponding to one beer can or 350 ml of draft beer, a 90 ml wine glass, a 30 ml dose of distilled beverage or one can or small bottle of any “iced” alcoholic beverage. Each of these doses contains approximately 10–12g of alcohol. The frequency was also asked in ranges, varying from everyday to never. By combining information on quantity and frequency of drinking wine, beer, liquor, and alcopops, respondents were classified into the following categories: Frequent heavy drinker (drinks once a week or more and has five or more drinks per occasion, at least once a week or more - a drink is 1 ounce of spirits, a 3-ounce glass of wine, or a 12-ounce can of beer); Frequent drinker (drinks once a week or more, but drinks five or more drinks per occasion less than once a week or not at all); Less frequent drinker (drinks one to three times a month, whether or not drinking five or more drinks per occasion); Infrequent drinker (drinks less than once a month but at least once a year, whether or not drinking five or more drinks per occasion); and Abstainer (drinks less than once a year or has never drunk alcohol).

d) Binge drinking

“During the past 12 months, how often have you drunk (men: five or more drinks or for women: four or more drinks) of any alcoholic beverage in one single occasion?” (Possible answers: from everyday to never in the past 12 months).

e) DSM-IV alcohol abuse

A positive answer to at least one of six questions on alcohol abuse of the Composite International Diagnostic Interview- Substance Abuse Module (CIDI-SAM) [32] in the past 12 months.

f) DSM-IV alcohol dependence

A positive answer to at least three of seven questions on dependence of the CIDI-SAM in the past 12 months.

#### Depressive symptoms

Depressive symptoms were evaluated using the Center for Epidemiologic Studies Depression Scale (CES-D) [33], which contains 20 items assessing current depressive symptoms in the general population, with an emphasis on depressive mood during the week preceding the assessment. CES-D scores range from 0 to 60, with higher scores indicating more depressive symptoms. The cut-off score of ≥16 is commonly used to discriminate persons at risk for depression. Scores between 16 and 26 indicate mild to moderate depression, and scores above 27 may be indicative of major depression [34], although a risk of overestimation has been detected in some studies [35]. High scores reflect the intensity of the discomfort that accompanies depression. Validation study of a CES-D Portuguese version in a sample of primary care patients in Brazil showed a satisfactory global performance, even though there were too many false-positive results. The area under the ROC curve (AUC) was 0.80, with a 91% sensitivity and 52% specificity, for the cut-off score of 18. This version also showed significant reliability, with an internal consistency of α = 0.90 [35]. Another study conducted among adolescents and young adults college students in Brazil found that the best performance of the CES-D was obtained when using cut-off score of 15. Using this cut-off value, the scale showed excellent sensitivity (1.0) with respect to the diagnoses of depressive disorders, a specificity of 0.75 and a misclassification rate of 0.24, with a reliability of α = 0.84 [36].

Finally, another Brazilian study found the best performance of the CES-D when using the cut-off score of 14 for high-school students [37]. Socioeconomic issues, cultural differences and the way people express their feelings may be important factors to determine the most appropriate cut-off point.

Overall, the scale has a high internal consistency [33] and a good level of agreement between the cut-off point of 16 or more (used in this article) and a clinical diagnosis of depression [38,39].

#### Socio-demographics

The variables assessed were: gender (male or female), age (six categories, see Table 1), marital status (single, married/partner, or divorced/separated/widowed), schooling (four categories, from illiterate/basic up to incomplete or complete higher education), family income (five categories, from ≤ $208 to > $1,157 US dollars (USD), or no information, because respondent did not know or refused to answer), Brazilian geographic regions (North, Mid-West, Northeast, or Southeast, South), and sector (rural or urban).

### Statistical analysis

The data were weighted to take into account the probability of the sample (and oversampling) selection and non-response rates. Data were initially weighted by an expansion factor which assigns to each individual the inverse of his/her probability of selection. This factor was multiplied by another weighting factor aimed at correcting for the non-response bias (for gender-, schooling- and regional-specific nonresponse rates). We also applied a post-stratification weight to adjust the sample (and oversampling) to known census-based distributions of the population for selected demographic variables (gender, age and region of the country) [40]. All analyses were performed with complex samples procedures using SVY commands from STATA 12.0 [41].

Associations in the bi-variate analyses (Tables 1 and 2) were tested using Rao-Scott measures as described elsewhere [42]. Gender specific multinomial logistic regression models were developed. The dependent variable, depressive symptoms, was divided into three categories: No Depressive Symptoms (reference), Mild/moderate, and Major. Relative risk ratios (RRR) were obtained by means of robust estimation, after weighting for sample design corrections, their 95% confidence intervals were computed, and a *p* value of 0.05 indicated significance.

**Table 2:** Characteristics of the study sample, by depressive symptoms

**Demographics and alcohol correlates**	**No depressive symptoms**		**Mild/Moderate**		**Major**		**Total**		***P-value***
	**(%)**	**(n)**	**(%)**	**(n)**	**(%)**	**(n)**	**(%)**	**(n)**	
**Gender**									
Male	80.6	1002	9.6	144	9.8	121	19.4	265	< 0.01
Female	62	1019	16.7	269	21.3	393	38	662	
**Age group**									
14 – 17	69.8	462	17	108	13.3	83	30.3	191	< 0.01
18 – 24	71.6	247	15	60	13.4	57	28.4	117	
25 – 34	72.9	408	12.1	68	15	103	27.1	171	
35 – 44	77.2	357	10.5	52	12.3	66	22.8	118	
45 – 59	68.1	314	11.6	60	20.2	117	31.8	177	
≥ 60	63.7	233	16.5	65	19.8	88	36.3	153	
**Marital status**									
Single	71.9	803	15	176	13.1	161	28.1	337	< 0.01
Married	73	997	11.8	177	15.2	240	27	417	
Divorced/Separeted/Widowed	58.7	221	15.5	60	25.8	113	41.3	173	
**Schooling**									
Illiterate/basic	64.3	604	14.3	152	21.4	228	35.7	380	< 0.01
Incomplete or complete elementary school	70.4	597	14.7	129	14.9	132	29.6	261	
Incomplete or complete high school	75.2	687	12.6	119	12.2	134	24.8	253	
Incomplete or complete higher education	84.9	133	7.3	13	7.8	20	15.1	33	
**Family income**									
≤ US $208	63.5	753	16.2	199	20.3	252	36.5	451	< 0.01
US $209 – US $347	68	384	15.2	86	16.8	102	32	188	
US $348 – US $555	76.6	355	11.5	55	11.9	67	23.4	122	
US $556 – US $1,157	79.7	231	8.5	24	11.8	44	20.3	68	
> US $1,157	84.1	97	8.7	14	7.2	9	15.9	23	
Not informed	77.6	201	10.2	35	12.2	40	22.4	75	
**Region**									
North	59	120	23.1	36	17.9	41	41	77	< 0.04
Mid-west	67.4	146	14.8	38	17.8	47	32.6	85	
Northeast	73	611	11.2	111	15.8	147	27	258	
Southeast	71.9	858	13	176	15.1	217	28.1	393	
South	73.8	286	11.7	52	14.5	62	26.2	114	
**Sector**									
Urban	71.7	1701	12.9	337	15.4	441	28.3	778	*0.39*
Rural	67.9	320	15.3	76	16.8	73	32.1	149	
**Age at first use**									
No drinking	63.3	854	16.6	230	20.1	298	36.7	528	< 0.01
Up to 14 years	69.5	223	11.4	43	19.1	51	30.5	94	
15 - 17 years	77.4	379	12.8	70	9.8	56	22.6	126	
18-24 years	79.8	424	9.6	54	10.6	72	20.2	126	
≥ 25 years	75.2	107	9	12	15.8	28	24.8	40	
**Problems related to alcohol consumption**									
None	70.6	1605	14	339	15.3	392	29.3	731	< 0.01
One	80.1	112	8.5	17	11.4	25	19.9	42	
Two	79.9	84	10.3	10	9.9	9	20.2	19	
Three	80.4	57	12.2	11	7.4	8	19.6	19	
≥ Four	64	163	11.9	36	24.1	80	36	116	
**Volume-Variability Index**									
Frequent heavy drinker	82.3	148	7.3	18	10.3	26	17.6	192	< 0.01
Frequent drinker	79.6	283	9.9	37	10.6	43	20.5	363	
Less frequent drinker	75.5	289	10.8	45	13.7	57	24.5	391	
Infrequent drinker	74.6	299	12.9	55	12.5	59	25.4	413	
Abstainer	64.5	1002	16	258	19.4	329	35.4	1589	
**Binge drinking**									
Abstainer	64.6	1002	16.1	258	19.3	328	35.4	586	< 0.01
No	78.8	508	10.2	68	11	83	21.2	151	
Yes	76.1	511	10.9	87	13	103	23.9	190	
**Abuse**									
No/Abstainer	71.7	1890	13.2	384	15.1	462	28.3	846	*0.08*
Yes	64.2	131	13.7	29	22.1	52	35.8	81	
**Dependence**									
No/Abstainer	72.6	1916	12.9	382	14.5	445	27.4	827	< 0.01
Yes	54	105	17.2	31	28.8	69	46	100	

## Results

1. Characteristics of the sample according to the presence of depressive symptoms is shown in Table 2.

Depressive symptoms were more prevalent in women, in those ≥60 years old, in divorced/separated/widowed individuals, in participants with illiteracy/basic education, with a family income ≤ $208 (USD), living in the Northern region, who first used alcohol when ≤14 years old, with alcohol dependence, with four or more problems related to alcohol consumption, in the abstainer group of the volume-variability index and with a binge-drinking pattern. Living in the rural sector and alcohol abuse were not significant.

Participants with alcohol dependence were found to have a higher prevalence of depressive symptoms, 46% (17.2% mild/moderate and 28.8% major/severe), compared to 27.4% in abstainers and those without alcohol dependence.

2. Mild/moderate depressive symptoms, multivariate results (Table 3).

**Table 3:** Results of the multivariate analysis through multinomial logistic regression for the variable “presence of depressive symptoms”, by genderª

	**Mild/Moderate depressive symptoms**				**Major depressive symptoms**			
	**Male (n = 1,161)**		**Female (n = 1,511)**		**Male (n = 1,161)**		**Female (n = 1,511)**	
Variables	RRR (95% CI)	*P-value*	RRR (95% CI)	*P-value*	RRR (95% CI)	*P-value*	RRR (95% CI)	*P-value*
Age group								
< 45 years	1.00		1.00		1.00		1.00	
≥ 45 years	1.27 (0.76, 2.11)	0.36	1.28 (0.89, 1.45)	0.18	2.67 (1.59, 4.48)	0.01	1.33 (0.97, 1.82)	0.08
Marital Status								
Married/Partner	1.00		1.00		1.00		1.00	
Single	1.52 (0.97, 2.40)	0.07	1.15 (0.82, 1.61)	0.42	1.30 (0.79, 2.14)	0.31	0.95 (0.69, 1.30)	0.74
Divorced/Separated/Widowed	1.84 (0.89, 3.81)	0.10	0.87 (0.56, 1.33)	0.51	1.86 (0.93, 3.72)	0.08	1.34 (0.94, 1.89)	0.10
Family income								
≥ US $341	1.00		1.00		1.00		1.00	
< US $340	1.42 (0.89, 2.27)	0.14	1.82 (1.27, 2.63)	0.01	1.59 (0.94, 2.69)	0.09	1.58 (1.15, 2.17)	0.01
Schooling								
> Elementary school	1.00		1.00		1.00		1.00	
≤ Elementary school	1.67 (1.03, 2.71)	0.04	1.12 (0.80, 1.57)	0.51	1.25 (0.73, 2.14)	0.42	1.25 (0.92, 1.69)	0.16
Region								
Southeast	1.00		1.00		1.00		1.00	
North	1.22 (0.63, 2.38)	0.55	1.30 (0.73, 2.29)	0.37	1.88 (0.87, 4.06)	0.11	0.96 (0.56, 1.65)	0.89
Mid-west	0.92 (0.42, 2.00)	0.83	1.36 (0.80, 2.31)	0.25	1.87 (0.86, 4.09)	0.12	1.11 (0.68, 1.80)	0.68
South	1.00 (0.56, 1.79)	0.10	0.75 (0.46, 1.22)	0.25	1.21 (0.61, 2.40)	0.59	0.71 (0.46, 1.08)	0.11
Northeast	0.44 (0.26, 0.74)	0.01	0.95 (0.67, 1.36)	0.80	1.21 (0.72, 2.07)	0.47	0.80 (0.58, 1.10)	0.17
Sector								
Urban	1.00		1.00		1.00		1.00	
Rural	1.22 (0.75, 2.01)	0.42	0.95 (0.64, 1.41)	0.80	0.66 (0.36, 1.20)	0.17	0.79 (0.54, 1.15)	0.21
Age at first use								
No drinking	1.00		1.00		1.00		1.00	
Up to 14 years	0.54 (0.25, 1.18)	0.12	1.06 (0.56, 2.01)	0.86	0.28 (0.11, 0.73)	0.01	0.73 (0.40, 1.34)	0.31
15 – 17 years	0.65 (0.32, 1.35)	0.25	1.03 (0.61, 1.73)	0.91	0.25 (0.10, 0.61)	0.01	0.48 (0.28, 0.82)	0.01
18 – 24	0.20 (0.09, 0.49)	0.01	0.87 (0.56, 1.37)	0.55	0.25 (0.11, 0.60)	0.01	0.64 (0.42, 0.97)	0.04
≥ 25 years	0.10 (0.01, 0.77)	0.03	0.63 (0.30, 1.32)	0.22	0.46 (0.15, 1.42)	0.18	0.59 (0.33, 1.07)	0.09
Problems related to alcohol								
none	1.00		1.00		1.00		1.00	
≥ 1	1.45 (0.75, 2.82)	0.27	1.29 (0.70, 2.36)	0.41	2.31 (1.09, 4.91)	0.03	3.00 (1.73, 5.21)	0.01
Volume-Variability Index								
Abstainer/Infrequent drinker	1.00		1.00		1.00		1.00	
Frequent heavy drinker	0.81 (0.33, 1.97)	0.64	0.18 (0.05, 0.70)	0.01	0.44 (0.18, 1.12)	0.08	0.27 (0.10, 0.75)	0.01
Frequent drinker	0.90 (0.44, 1.86)	0.78	0.52 (0.27, 0.99)	0.04	0.88 (0.42, 1.86)	0.74	0.47 (0.25, 0.90)	0.02
Less frequent drinker	0.93 (0.43, 2.01)	0.86	0.69 (0.40, 1.17)	0.17	0.91(0.40, 2.05)	0.82	0.89 (0.54, 1.49)	0.67
Binge drinking								
No	1.00		1.00		1.00		1.00	
Yes	1.10 (0.59, 2.05)	0.29	1.18 (0.70, 1.99)	0.54	1.44 (0.74, 2.80)	0.29	0.56 (0.32, 0.96)	0.04
Abuse								
No	1.00		1.00		1.00		1.00	
Yes	1.38 (0.66, 2.90)	0.40	0.56( 0.17, 1.80)	0.33	1.89 (0.93, 3.87)	0.08	0.61 (0.23, 1.62)	0.32
Alcohol dependence								
No	1.00		1.00		1.00		1.00	
Yes	1.47 (0.67, 3.20)	0.33	4.09 (1.37, 12.17)	0.01	3.93 (1.88, 8.19)	0.01	8.58 (3.38, 21.75)	0.01

ªReference category: “No depressive symptoms”.

There were significant interactions of alcohol dependence and having one or more problems related to alcohol consumption with sex, thus all of the following results are broken down for males and females (see Additional file 1).

Among women, higher risks of mild/moderate depressive symptoms were observed in those earning less than $340 USD (2.5 times the minimum wage). Alcohol dependence multiplied the risk of depressive symptoms by 4.09 relative to women who abstained or were not dependent. Frequent heavy drinkers and frequent drinkers had lower risks of mild/moderate depressive symptoms. Alcohol abuse was not significant.

Among men, those with an educational level of elementary school or less had a higher risk of mild/moderate depressive symptoms. Living in the Northeast region and being 18-24 or ≥25 years old when alcohol use began were associated with lower risks of mild/moderate depressive symptoms. Alcohol abuse, alcohol dependence, being a frequent heavy drinker, and a binge-drinking pattern were not significant.

3. Major depressive symptoms, multivariate results (Table 3).

Among women, earning less than $340 (USD) was associated with a higher risk of major depressive symptoms. Having one or more problems related to alcohol consumption multiplied the risk of major depressive symptoms by 3.00 relative to women with no problems, and alcohol dependence multiplied this risk by 8.58 relative to no alcohol dependence. Being 15–17 or 18–24 years old when alcohol use began, frequent heavy drinking or frequent drinking, and a binge-drinking pattern were associated with lower risks of major depressive symptoms. Alcohol abuse was not significant.

Among men, age ≥45 years multiplied the risk of major depressive symptoms by 2.67, having one or more problems related to alcohol consumption multiplied this risk by 2.31, and alcohol dependence conferred a 3.93 times higher risk of major depressive symptoms. Finally, being ≤14, 15–17 or 18–24 years old when alcohol use began was associated with a lower risk of major depressive symptoms. Alcohol abuse, being a frequent heavy drinker, and a binge drinking pattern were not significant.

## Discussion

This study found the prevalence of depressive symptoms in Brazil to be higher in participants with alcohol dependence than in those not alcohol dependent and abstainers; that finding is consistent with other surveys worldwide [18,19]. Both mild/moderate and major depressive symptoms were more prevalent in alcohol dependent women, consistent with patterns observed in many other epidemiologic studies [20,21]. Alcohol dependent men also had high rates of major depressive symptoms. Based on our findings, we speculate that almost half of the alcohol dependent population experiences depressive symptoms and that alcohol dependence is most strongly associated with major depressive symptoms in women. This illustrates the strong relationship between depressive symptoms and alcohol dependence in Brazil. This dependence potentially represents a large socio-economic burden to individuals, especially to women, and to society [5]. In addition, this strong association between depressive symptoms and alcohol dependence corroborates data from the WHO about the possible impact of these conditions on YLD, especially in men [1]. This impact could threaten the socio-economic development of Brazil and may serve as a warning to other middle-income countries.

As expected, having four or more problems related to alcohol consumption was associated with the highest rates of depressive symptoms. Men and women with one or more problems had higher risks of major depressive symptoms than those without problems, with a slightly higher risk in women. These findings support the association between biopsychosocial distress, alcohol consumption and depressive symptoms, which reinforces the need for longitudinal studies aimed at establishing causal relationships between these conditions [12]. As mentioned above, the São Paulo metropolitan region study also found a positive association between depression and the occurrence of problems caused by alcohol use in men, but not in women [25].

Several socio-demographic characteristics were significantly associated with depressive symptoms. In men, a higher rate of major depressive symptoms was found in those ≥45 years old and mild/moderate depressive symptoms were more prevalent in those with lower educational levels. This is disturbing, because the elderly population in Brazil is increasing [43] and only a small fraction of the population has access to higher education [44]. In women, prevalence rates of depressive symptoms were inversely related to family income; that is, the lower the financial resources available, the higher the prevalence of these symptoms, consistent with previous investigations [45].

Studies have reported that a greater frequency of depressed mood during childhood was significantly associated with a younger onset of alcohol use, problems with alcohol use in adolescence, and adult alcohol dependence [46]. Our results show that initiation of alcohol use at ≤14 years of age was associated with the highest rates of depressive symptoms among people who drink. However, based on the regression models, the risks of depressive symptoms were lower among all age ranges of first alcohol use than in people who never drink, indicating that depressive symptoms were more prevalent in abstainers.

A similar effect of abstainers was observed in our analysis of binge drinking. Depressive symptoms were more prevalent in abstainers, and people who binge drink had a slightly higher prevalence of depressive symptoms than people who do not binge drink. These findings suggest an inverted J-shaped curve relationship. However, based on the regression models, the only significant association between major depressive symptoms and binge drinking was found in women, and binge drinkers had a lower risk than did women who do not drink in a binge pattern. Other studies also found mixed and contradictory findings regarding the association of depression and binge drinking [17], and some found gender differences in this relationship [16]. The São Paulo metropolitan region study reported a positive association between depression and binge drinking in men, but not in women [25].

Surprisingly, frequent heavy and frequent drinkers had the lowest rates of depressive symptoms, which is inconsistent with the results of other surveys [15,47]. Our results show that women who abstain and women who are infrequent drinkers had higher risks of depressive symptoms than women who are frequent heavy and frequent drinkers. These findings possibly contradict the J-shaped or U-shaped relationship mentioned above. Finally, our findings differed from an observation of the São Paulo metropolitan region study, which reported that male moderate drinkers had more prevalent depression than did mild and heavy drinkers [25].

These negative associations of depressive symptoms with the volume-variability index and the binge drinking pattern in women suggest that Brazilian women with depressive symptoms may not turn to alcohol to cope with a negative affect, which is inconsistent with other studies [14,48]. Alternatively, these negative associations may suggest that Brazilians who are frequent heavy drinkers or frequent drinkers do not suffer from depressive symptoms. These speculations do not support causal theories for the association between heavy drinking or alcohol use/abuse and depressive symptoms in the short-term [8]. These findings could lead an erroneous perception that heavy alcohol drinking does not cause depressive symptoms and to permissive alcohol use in the Brazilian population. However, the strong consistent associations, described above, between depressive symptoms and both alcohol dependence and problems related to alcohol consumption further support the association of biopsychosocial distress, long-term alcohol consumption and depressive symptoms [13]. Longitudinal studies in Brazil are necessary to address these issues more accurately, and relevant information should be given to the population to better prevent alcohol misuse that possibly leads to depression in the long-term.

The only socio-demographic characteristic that was significantly associated with a lower risk of depressive symptoms was the Northeast region. Men living in this region of Brazil had a lower risk of mild/moderate depressive symptoms than did men living in the Southeast region. This result was intriguing, because the Northeastern region of Brazil has limited social, economic, and health resources. Future multicenter surveys should include states in this region to investigate this issue in depth and to detect potential protective factors against depression in this region.

Some limitations of the present study should be mentioned. First, the cross-sectional design allowed measurement of the associations between depressive symptoms, alcohol correlates, and socio-demographic variables, but not the interpretation of causality. In addition, the CES-D is a depression screening instrument (rather than a diagnostic instrument) that assesses symptoms that occurred during the week before the assessment, which is not consistent with criteria to define major depression. Therefore, our results have limited comparability with studies that used more acceptable diagnostic measures of depression. This could even explain some of the negative associations described above, because another study [16] found that the overall relationship between depression and alcohol consumption is stronger for women than for men only when depression is measured using a clinical diagnosis of major depression and not when measured as a recent depressed affect. Furthermore, the CES-D cannot distinguish alcohol-induced depressive symptoms from depressive symptoms that occurred during the week before the assessment, which may cause false-positives using this screening instrument. Another limitation is the fact that our results are based on the 2000 national socio-demographic census [40]. In addition, the non-response rate was relatively high, although the sample is considered representative of the Brazilian population.

## Conclusion

We can infer from our results that the prevalence of depressive symptoms is strongly associated with alcohol dependence in Brazil. Major depressive symptoms are more frequent, and depressive symptoms rates are higher among women with low income and with one or more problems related to alcohol consumption. Among men, depressive symptoms rates are higher in the older age groups and in those who had one or more problems related to alcohol consumption. Men with limited education also had higher rates of mild/moderate depressive symptoms.

This study, which considered the biopsychosocial model of mental disorders, found that depression prevalence rates in Brazil are higher in certain groups, such as women, the elderly, and people with lower education and income, as well as in people with problems related to alcohol consumption and alcohol dependence. Therefore, this survey supports the possible association of biopsychosocial distress, alcohol consumption and the prevalence of depressive symptoms in Brazil.

However, it is important to mention that abstainers also had high rates of depressive symptoms. The lower risks of depressive symptoms associated with volume-variability index, binge drinking, and age at first use of alcohol reinforces the need to consider abstainers, former drinkers, lifetime abstainers, and other drinking patterns in general population studies of the association between alcohol use and depression [49].

In conclusion, this study provides the first evaluation of the prevalence of depressive symptoms in relation to alcohol correlates in Brazil. We included subgroups of the population at greatest risk for depression. These findings are potentially important because they are based on a large random sample of respondents and they may help to develop targeted programs aimed at assisting these groups. Further cross-sectional studies are needed to better understand risk and protective factors. Longitudinal studies aimed at establishing causal relationships could also help to develop coordinated and adequate interventions. Our data also suggest that investments in education, social programs, and care for those with alcohol dependence and major/severe depressive symptoms, especially women, together with the development of alcohol prevention policies, may support a strategic plan to reduce the prevalence of depression and alcohol-related problems in Brazil.

Finally, a better understanding of these associations may also promote the socio-economic development of Brazil and other middle-income countries.

## Competing interests

JASC and JLFS receive CNPq Productivity Awards (Brazil). All other authors declare that they have no competing interests.

## Authors’ contributions

CLSC participated in the analysis and interpretation of data and drafted the manuscript. JASC participated in the analysis and interpretation of data and critically revised the intellectual content of the manuscript. JLFS performed the statistical analysis, participated in the interpretation of data and critically revised the intellectual content of the manuscript. IP participated in the conception, design, and acquisition of data and critically revised the intellectual content of the manuscript. MZ participated in the conception, design and acquisition of data and critically revised the intellectual content of the manuscript. RC participated in the conception, design, and acquisition of data and critically revised the intellectual content of the manuscript. RRL participated in the conception, design, and acquisition of data and critically revised the intellectual content of the manuscript. All authors read and approved the final manuscript.

## Supplementary Material

Additional file 1 Regression both sex & interactions. Click here for file
