# Supplementary material for: Depressive symptoms and alcohol correlates among Brazilians aged 14 years and older: a cross-sectional study
Source: Subst Abuse Treat Prev Policy. 2014 Jul 15;9:29. doi: 10.1186/1747-597X-9-29 (PMC4105397; doi:10.1186/1747-597X-9-29)
Supplement: Additional file 1 — Regression both sex & interactions. [file 1747-597X-9-29-S1.docx]

**REGRESSION BOTH SEX & INTERACTIONS**

**log type: text**

**opened on: 4 Apr 2014, 15:50:40**

**. mlogit depress3 catage2 single sepdivwid schooling famincome north mid northeasth south sectrur firstuse14 firstuse15 firstuse18 firstuse25 problems freqheav freq lessfreq binge2 abuse alcdep2 sex,rrr**

**Iteration 0: log likelihood = -2257.8372**

**Iteration 1: log likelihood = -2077.8129**

**Iteration 2: log likelihood = -2067.0384**

**Iteration 3: log likelihood = -2066.9957**

**Iteration 4: log likelihood = -2066.9957**

**Multinomial logistic regression Number of obs = 2672**

**LR chi2(44) = 381.68**

**Prob > chi2 = 0.0000**

**Log likelihood = -2066.9957 Pseudo R2 = 0.0845**

**-------------------------------------------------------------------------------**

**depress3 | RRR Std. Err. z P>|z| [95% Conf. Interval]**

**--------------+----------------------------------------------------------------**

**Sem | (base outcome)**

**--------------+----------------------------------------------------------------**

**Mild_Moderate |**

**catage2 | 1.258731 .1864901 1.55 0.120 .9415017 1.682847**

**single | 1.301374 .1760023 1.95 0.051 .9983497 1.696374**

**sepdivwid | 1.04195 .195843 0.22 0.827 .7208696 1.506042**

**schooling | 1.276886 .1770287 1.76 0.078 .9730627 1.675574**

**famincome | 1.662286 .2417912 3.49 0.000 1.24995 2.210645**

**north | 1.268088 .2781991 1.08 0.279 .8249142 1.949351**

**mid | 1.2279 .2711569 0.93 0.353 .7965121 1.892926**

**northeast | .7454882 .1097204 -2.00 0.046 .5586779 .9947638**

**south | .8581739 .1625417 -0.81 0.419 .5920443 1.243931**

**sectrur | 1.035554 .161976 0.22 0.823 .7621352 1.407062**

**firstuse14 | .8574079 .2053732 -0.64 0.521 .536168 1.371116**

**firstuse15 | .9371426 .1936305 -0.31 0.753 .6250751 1.405009**

**firstuse18 | .5896497 .1172801 -2.66 0.008 .3992932 .8707556**

**firstuse25 | .4531129 .1561155 -2.30 0.022 .2306394 .8901831**

**problems | 1.235336 .2666287 0.98 0.327 .8092193 1.885837**

**freqheav | .4431649 .1511657 -2.39 0.017 .2270997 .8647972**

**freq | .6166639 .146523 -2.03 0.042 .3870775 .9824244**

**lessfreq | .7391214 .1622034 -1.38 0.168 .4807463 1.136359**

**binge2 | 1.180448 .2358384 0.83 0.406 .7979719 1.746248**

**abuse | 1.099265 .3374835 0.31 0.758 .6022496 2.00645**

**alcdep2 | 2.044129 .6431396 2.27 0.023 1.103308 3.787214**

**sex | 2.14243 .275694 5.92 0.000 1.664837 2.757031**

**_cons | .0845428 .0171435 -12.18 0.000 .056816 .1258007**

**--------------+----------------------------------------------------------------**

**Depress_Major |**

**catage2 | 1.565479 .2094554 3.35 0.001 1.204369 2.034862**

**single | 1.018949 .1370806 0.14 0.889 .782779 1.326373**

**sepdivwid | 1.378546 .216213 2.05 0.041 1.01372 1.87467**

**schooling | 1.257259 .1673862 1.72 0.086 .9684995 1.632113**

**famincome | 1.58065 .2164154 3.34 0.001 1.208629 2.06718**

**north | 1.144618 .2550762 0.61 0.544 .7395573 1.771534**

**mid | 1.268176 .2643931 1.14 0.254 .8427887 1.908274**

**northeast | .8531891 .1179605 -1.15 0.251 .6506683 1.118745**

**south | .8347376 .1517814 -0.99 0.320 .5844871 1.192134**

**sectrur | .7678414 .1230396 -1.65 0.099 .5608855 1.05116**

**firstuse14 | .5765542 .141465 -2.24 0.025 .3564403 .9325957**

**firstuse15 | .4324317 .0974936 -3.72 0.000 .277978 .6727049**

**firstuse18 | .5010385 .0939309 -3.69 0.000 .3469719 .7235155 f firstuse25 | .5845651 .1548113 -2.03 0.043 .3478627 .9823312**

**problems | 2.46079 .5261154 4.21 0.000 1.618409 3.741631**

**freqheav | .389912 .12806 -2.87 0.004 .2048349 .7422143**

**freq | .6035006 .1440418 -2.12 0.034 .3780205 .9634741**

**lessfreq | .8749823 .1891672 -0.62 0.537 .5727608 1.336673**

**binge2 | .7954125 .1613617 -1.13 0.259 .5344574 1.183782**

**abuse | 1.260833 .3531239 0.83 0.408 .728216 2.183008**

**alcdep2 | 4.973119 1.396947 5.71 0.000 2.867653 8.624442**

**sex | 3.815595 .5137027 9.95 0.000 2.930643 4.967771**

**_cons | .0704368 .0142169 -13.14 0.000 .0474234 .1046178**

**INTERACTION SEX X ALCOHOL DEPENDENCE**

**. mlogit depress3 catage2 single sepdivwid schooling famincome north mid northeasth south sectrur firstuse14 firstuse15 firstuse18 firstuse25 problems freqheav freq lessfreq binge2 abuse alcdep2 sexalcdep,rrr**

**Iteration 0: log likelihood = -2257.8372**

**Iteration 1: log likelihood = -2139.2419**

**Iteration 2: log likelihood = -2132.9981**

**Iteration 3: log likelihood = -2132.9493**

**Iteration 4: log likelihood = -2132.9493**

**Multinomial logistic regression Number of obs = 2672**

**LR chi2(42) = 249.78**

**Prob > chi2 = 0.0000**

**Log likelihood = -2132.9493 Pseudo R2 = 0.0553**

**-------------------------------------------------------------------------------**

**depress3 | RRR Std. Err. z P>|z| [95% Conf. Interval]**

**--------------+----------------------------------------------------------------**

**No | (base outcome)**

**--------------+----------------------------------------------------------------**

**Mild_Moderate |**

**catage2 | 1.188104 .174591 1.17 0.241 .8907814 1.584667**

**single | 1.222579 .1643203 1.50 0.135 .939445 1.591045**

**sepdivwid | 1.190698 .2202214 0.94 0.345 .8286491 1.710931**

**instruction | 1.202084 .1645152 1.34 0.179 .9192654 1.571915**

**famincome | 1.725124 .2492311 3.77 0.000 1.299709 2.289784**

**north | 1.251058 .2721556 1.03 0.303 .8167836 1.916233**

**mid | 1.269305 .2781836 1.09 0.277 .8260664 1.950371**

**northeast | .7618507 .1113347 -1.86 0.063 .5721075 1.014523**

**south | .8736471 .1643348 -0.72 0.473 .6042592 1.263132**

**sectrur | 1.014438 .157506 0.09 0.926 .7482799 1.375266**

**firstuse14 | .8390228 .1986473 -0.74 0.458 .5275249 1.334457**

**firstuse15 | .9133733 .1874622 -0.44 0.659 .6108666 1.365684**

**firstuse18 | .5970274 .1180189 -2.61 0.009 .4052574 .8795441**

**firstuse25 | .4860378 .1666804 -2.10 0.035 .2481769 .9518724**

**problems | 1.098441 .2266552 0.46 0.649 .733057 1.645948**

**freqheav | .4272998 .1425411 -2.55 0.011 .2222204 .8216396**

**freq | .5755479 .1352272 -2.35 0.019 .363152 .9121674**

**lessfreq | .7469871 .1627201 -1.34 0.181 .4874066 1.144814**

**binge2 | 1.168026 .231493 0.78 0.433 .7920483 1.722478**

**abuse | 1.252704 .3395632 0.83 0.406 .7364077 2.130977**

**sexalcdep | 3.317103 1.415017 2.81 0.005 1.437645 7.653609**

**_cons | .1432107 .0251091 -11.08 0.000 .1015627 .2019372**

**--------------+----------------------------------------------------------------**

**Depress_Major |**

**catage2 | 1.428724 .1864619 2.73 0.006 1.106263 1.845177**

**single | .9317373 .1230773 -0.54 0.592 .719208 1.20707**

**sepdivwid | 1.701963 .2578974 3.51 0.000 1.264643 2.29051**

**instruction | 1.159784 .1503329 1.14 0.253 .8995865 1.49524**

**famincome | 1.657086 .2216953 3.78 0.000 1.27487 2.153893**

**north | 1.12819 .2454512 0.55 0.579 .7365347 1.72811**

**mid | 1.322377 .2697579 1.37 0.171 .8865732 1.972404**

**northeast | .888251 .1198485 -0.88 0.380 .6818458 1.157138**

**soutth | .8548431 .1525821 -0.88 0.380 .6024961 1.212882**

**sectrur | .7434973 .11649 -1.89 0.059 .5469083 1.010751**

**firstuse14 | .5622272 .133542 -2.42 0.015 .3529651 .8955542**

**firstuse15 | .4018788 .0889164 -4.12 0.000 .2604745 .6200477**

**firstuse18 | .4781888 .0885943 -3.98 0.000 .3325806 .6875464**

**firstuse25 | .6276413 .1637209 -1.79 0.074 .3764217 1.046522**

**problems | 2.097467 .4123231 3.77 0.000 1.426805 3.083369**

**freqheav | .3864308 .1224396 -3.00 0.003 .2076685 .7190726**

**freq | .5549176 .1277465 -2.56 0.011 .353407 .8713285**

**lessfreq | .9089152 .1912029 -0.45 0.650 .6018127 1.372731**

**binge2 | .8478199 .1659579 -0.84 0.399 .5776753 1.244295**

**abuse | 1.818053 .4347758 2.50 0.012 1.13775 2.905132**

**sexalcdep | 6.901133 2.376653 5.61 0.000 3.513812 13.55384**

**_cons | .1909993 .030806 -10.26 0.000 .1392332 .2620117**

**-------------------------------------------------------------------------------**

**. ************ INTERACTION SEX X FREQUENT DRINKER *****************

**. mlogit depress3 catage2 single sepdivwid schooling famincome north mid northeasth south sectrur firstuse14 firstuse15 firstuse18 firstuse25 problems freqheav lessfreq binge2 abuse alcdep2 sexfreq,rrr**

**Iteration 0: log likelihood = -2257.8372**

**Iteration 1: log likelihood = -2142.6472**

**Iteration 2: log likelihood = -2137.5876**

**Iteration 3: log likelihood = -2137.5664**

**Iteration 4: log likelihood = -2137.5664**

**Multinomial logistic regression Number of obs = 2672**

**LR chi2(42) = 240.54**

**Prob > chi2 = 0.0000**

**Log likelihood = -2137.5664 Pseudo R2 = 0.0533**

**-------------------------------------------------------------------------------**

**depress3 | RRR Std. Err. z P>|z| [95% Conf. Interval]**

**--------------+----------------------------------------------------------------**

**No | (base outcome)**

**--------------+----------------------------------------------------------------**

**Mild_Moderate |**

**catage2 | 1.171964 .171711 1.08 0.279 .8794272 1.561811**

**single | 1.233865 .165431 1.57 0.117 .9487296 1.604697**

**sepdivwid | 1.209036 .2229376 1.03 0.303 .8423325 1.73538**

**instruction | 1.17971 .1610763 1.21 0.226 .9027205 1.541691**

**famincome | 1.736965 .2503391 3.83 0.000 1.30952 2.303934**

**north | 1.255812 .2727517 1.05 0.294 .8204475 1.922199**

**mid | 1.271594 .2783654 1.10 0.272 .8279642 1.952925**

**northeast | .7613195 .1111056 -1.87 0.062 .5719316 1.013421**

**south | .8664987 .1626055 -0.76 0.445 .5998366 1.251707**

**sectrur | 1.010178 .1566302 0.07 0.948 .7454479 1.368921**

**firstuse14 | .7101557 .1659099 -1.47 0.143 .4492539 1.122575**

**firstuse15 | .7808696 .1566783 -1.23 0.218 .5269731 1.157094**

**firstuse18 | .5201895 .1015912 -3.35 0.001 .3547521 .7627782**

**firstuse25 | .426291 .1452197 -2.50 0.012 .2186437 .8311422**

**problems | 1.017302 .2135772 0.08 0.935 .6741312 1.535165**

**freqheav | .5468451 .1740325 -1.90 0.058 .2930698 1.02037**

**lessfreq | .9430388 .1928497 -0.29 0.774 .6316267 1.407987**

**binge2 | 1.045069 .2015394 0.23 0.819 .7161315 1.525097**

**abuse | .9554471 .2881407 -0.15 0.880 .5290589 1.725478**

**alcdep2 | 1.975611 .6110753 2.20 0.028 1.077498 3.622315**

**sexfreq | 1.083235 .317721 0.27 0.785 .6096197 1.924804**

**_cons | .1441258 .0252291 -11.07 0.000 .1022679 .2031159**

**--------------+----------------------------------------------------------------**

**Depress_Major |**

**catage2 | 1.390628 .1807071 2.54 0.011 1.077955 1.793996**

**single | .9375604 .1233616 -0.49 0.624 .7244368 1.213383**

**sepdivwid | 1.759013 .265371 3.74 0.000 1.308741 2.3642**

**instruction | 1.113488 .1439266 0.83 0.406 .8642941 1.43453**

**famincome | 1.662618 .2218721 3.81 0.000 1.279976 2.159648**

**north | 1.112171 .2423625 0.49 0.626 .7255695 1.704763**

**mid | 1.323598 .2693741 1.38 0.168 .8882244 1.972376**

**northeast | .8735685 .1178442 -1.00 0.316 .6706101 1.137952**

**south | .8615452 .1525939 -0.84 0.400 .6088582 1.219102**

**sectrur | .7376421 .1155735 -1.94 0.052 .5425999 1.002794**

**firstuse14 | .4411393 .1046074 -3.45 0.001 .2771599 .7021359**

**firstuse15 | .3273234 .0712211 -5.13 0.000 .2136816 .501403**

**firstuse18 | .4189249 .0763419 -4.77 0.000 .2931023 .5987606**

**firstuse25 | .5598184 .1430933 -2.27 0.023 .3392145 .9238892**

**problems | 1.74001 .3531162 2.73 0.006 1.168987 2.589965**

**freqheav | .4809046 .1430961 -2.46 0.014 .2683976 .8616663**

**lessfreq | 1.171192 .2295487 0.81 0.420 .7976199 1.719729**

**binge2 | .751683 .145538 -1.47 0.140 .514314 1.098604**

**abuse | .9970265 .268054 -0.01 0.991 .5886516 1.68871**

**alcdep2 | 4.579442 1.229609 5.67 0.000 2.705576 7.751138**

**sexfreq | 1.132672 .3288961 0.43 0.668 .6411197 2.001102**

**_cons | .1975398 .0316787 -10.11 0.000 .1442615 .2704945**

**-------------------------------------------------------------------------------**

**. *************** INTERACTION SEX X FREQUENT HEAVY **************

**. mlogit depress3 catage2 single sepdivwid schooling famincome north mid northeasth south sectrur firstuse14 firstuse15 firstuse18 firstuse25 problems freq lessfreq binge2 abuse alcdep2 sexfreqheav,rrr**

**Iteration 0: log likelihood = -2257.8372**

**Iteration 1: log likelihood = -2144.5244**

**Iteration 2: log likelihood = -2139.5689**

**Iteration 3: log likelihood = -2139.548**

**Iteration 4: log likelihood = -2139.548**

**Multinomial logistic regression Number of obs = 2672**

**LR chi2(42) = 236.58**

**Prob > chi2 = 0.0000**

**Log likelihood = -2139.548 Pseudo R2 = 0.0524**

**-------------------------------------------------------------------------------**

**depress3 | RRR Std. Err. z P>|z| [95% Conf. Interval]**

**--------------+----------------------------------------------------------------**

**No | (base outcome)**

**--------------+----------------------------------------------------------------**

**Mild_Moderate |**

**catage2 | 1.168435 .1714896 1.06 0.289 .8763445 1.557881**

**single | 1.2381 .1659321 1.59 0.111 .9520866 1.610034**

**sepdivwid | 1.22298 .2261822 1.09 0.276 .8511283 1.75729**

**instruction | 1.182675 .161489 1.23 0.219 .9049773 1.545586**

**famincome | 1.720662 .2479187 3.77 0.000 1.297333 2.282125**

**north | 1.229481 .2668315 0.95 0.341 .8035032 1.881292**

**mid | 1.268254 .2777071 1.09 0.278 .8256961 1.948014**

**northeast | .742663 .1083861 -2.04 0.041 .5579115 .9885946**

**south | .8680325 .1628676 -0.75 0.451 .6009334 1.25385**

**sectrur | 1.030056 .1596946 0.19 0.849 .7601426 1.395812**

**firstuse14 | .7781581 .1838627 -1.06 0.288 .4897185 1.236486**

**firstuse15 | .8358987 .1703543 -0.88 0.379 .5606356 1.246312**

**firstuse18 | .5712465 .1130887 -2.83 0.005 .3875365 .8420433**

**firstuse25 | .4650819 .1590328 -2.24 0.025 .2379385 .9090633**

**problems | .9824817 .2059706 -0.08 0.933 .6514421 1.481744**

**freq | .6939755 .1525122 -1.66 0.096 .4511065 1.067602**

**lessfreq | .8842688 .182252 -0.60 0.551 .5904018 1.324405**

**binge2 | 1.050317 .201616 0.26 0.798 .7209848 1.530082**

**abuse | .9087548 .2748313 -0.32 0.752 .5023669 1.643889**

**alcdep2 | 1.90217 .5865587 2.09 0.037 1.03937 3.481195**

**sexfreqheav | .4418882 .2780362 -1.30 0.194 .1287468 1.516661**

**_cons | .1456314 .0255043 -11.00 0.000 .1033201 .2052697**

**--------------+----------------------------------------------------------------**

**Depress_Major |**

**catage2 | 1.395414 .1816239 2.56 0.010 1.081216 1.800916**

**single | .9433986 .1238293 -0.44 0.657 .7294031 1.220177**

**sepdivwid | 1.753139 .2653091 3.71 0.000 1.303169 2.358478**

**instruction | 1.11873 .1443614 0.87 0.385 .868732 1.440671**

**famincome | 1.633812 .2173496 3.69 0.000 1.258824 2.120505**

**north | 1.087279 .2367083 0.38 0.701 .7096245 1.665918**

**mid | 1.333734 .2714777 1.41 0.157 .8949722 1.987599**

**northeast | .859311 .1157723 -1.13 0.260 .6598886 1.119**

**south | .8652753 .1531 -0.82 0.413 .6117084 1.223951**

**sectrur | .7540695 .1179858 -1.80 0.071 .5549166 1.024696**

**firstuse14 | .4735255 .1135532 -3.12 0.002 .2959526 .7576431**

**firstuse15 | .342129 .0755937 -4.85 0.000 .2218789 .5275501**

**firstuse18 | .4520686 .0839282 -4.28 0.000 .314178 .6504785**

**firstuse25 | .5915464 .1523607 -2.04 0.042 .3570676 .9800026**

**problems | 1.671588 .3395142 2.53 0.011 1.122646 2.488948**

**freq | .7179646 .1535615 -1.55 0.121 .4721111 1.091847**

**lessfreq | 1.166724 .2298503 0.78 0.434 .7930075 1.71656**

**binge2 | .7172939 .1366404 -1.74 0.081 .4937967 1.041948**

**abuso | .9426745 .2511786 -0.22 0.825 .5591862 1.589158**

**alcdep2 | 4.370773 1.161588 5.55 0.000 2.596215 7.35827**

**sexfreqheav | .8161332 .3674958 -0.45 0.652 .3376559 1.97264**

**_cons | .1998942 .0320197 -10.05 0.000 .1460332 .2736205**

**-------------------------------------------------------------------------------**

**Total | 3,007 100.00**

**. gen sexprob = sex*problems**

**. ************** INTERACTION SEX X PROBLEMS ****************

**. mlogit depress3 catage2 single sepdivwid schooling famincome north mid northeasth south sectrur firstuse14 firstuse15 firstuse18 firstuse25 freqpes freq lessfreq binge2 abuse alcdep2 sexfprob,rrr**

**Iteration 0: log likelihood = -2257.8372**

**Iteration 1: log likelihood = -2123.4031**

**Iteration 2: log likelihood = -2116.5704**

**Iteration 3: log likelihood = -2116.5379**

**Iteration 4: log likelihood = -2116.5379**

**Multinomial logistic regression Number of obs = 2672**

**LR chi2(42) = 282.60**

**Prob > chi2 = 0.0000**

**Log likelihood = -2116.5379 Pseudo R2 = 0.0626**

**-------------------------------------------------------------------------------**

**depress3 | RRR Std. Err. z P>|z| [95% Conf. Interval]**

**--------------+----------------------------------------------------------------**

**No | (base outcome)**

**--------------+----------------------------------------------------------------**

**Mild_Moderate |**

**catage2 | 1.208504 .1779804 1.29 0.198 .905501 1.6129**

**single | 1.230773 .1650989 1.55 0.122 .9462278 1.600885**

**sepdivwid | 1.177868 .2177977 0.89 0.376 .8197895 1.692353**

**instruction | 1.21227 .1666173 1.40 0.161 .9259944 1.58705**

**famincome | 1.684077 .2437449 3.60 0.000 1.268128 2.236457**

**north | 1.255033 .2731477 1.04 0.297 .8192155 1.922703**

**mid | 1.26109 .2766012 1.06 0.290 .8204421 1.938404**

**northeast | .765601 .1120095 -1.83 0.068 .5747373 1.019848**

**south | .8876491 .1670257 -0.63 0.526 .6138664 1.283538**

**sectrur | 1.022743 .1589279 0.14 0.885 .754215 1.386877**

**firstuse14 | .7917439 .1840099 -1.00 0.315 .5020607 1.248571**

**firstuse15 | .8978078 .1806071 -0.54 0.592 .6052737 1.331726**

**firstuse18 | .5888929 .1161494 -2.68 0.007 .4000837 .8668058**

**firstuse25 | .4723659 .1623773 -2.18 0.029 .2408104 .9265775**

**freqheav | .4029979 .1350485 -2.71 0.007 .208957 .7772283**

**freq | .567885 .1335124 -2.41 0.016 .3582111 .9002888**

**lessfreq | .7324192 .1600354 -1.43 0.154 .4772765 1.123956**

**binge2 | 1.099026 .2154326 0.48 0.630 .7484356 1.613845**

**abuse | 1.03204 .310444 0.10 0.917 .5723344 1.860986**

**alcdep2 | 1.955342 .592411 2.21 0.027 1.079776 3.540884**

**sexprob | 1.949596 .4736033 2.75 0.006 1.211065 3.138497**

**_cons | .1432124 .0251264 -11.08 0.000 .1015404 .2019865**

**--------------+----------------------------------------------------------------**

**Depress_Major |**

**catage2 | 1.493403 .1968286 3.04 0.002 1.153426 1.933589**

**single | .9231734 .122438 -0.60 0.547 .7118537 1.197225**

**sepdivwid | 1.634315 .248724 3.23 0.001 1.21281 2.202312**

**instruction | 1.171211 .153477 1.21 0.228 .905926 1.514181**

**famincome | 1.610234 .2171996 3.53 0.000 1.236155 2.097515**

**north | 1.134216 .2477956 0.58 0.564 .7391474 1.740445**

**mid | 1.329604 .2730107 1.39 0.165 .8890849 1.98839**

**northeast | .8896201 .1208208 -0.86 0.389 .6817128 1.160935**

**south | .8744661 .1566306 -0.75 0.454 .6155728 1.242243**

**sectrur | .7487104 .1180034 -1.84 0.066 .5497396 1.019696**

**firstuse14 | .5678115 .1325191 -2.43 0.015 .3593733 .8971447**

**firstuse15 | .4312132 .0934571 -3.88 0.000 .281975 .6594373**

**firstuse18 | .5096299 .0932707 -3.68 0.000 .3560171 .7295228**

**firstuse25 | .6264852 .1643654 -1.78 0.075 .3746179 1.047691**

**freqheav | .3615956 .1174018 -3.13 0.002 .1913628 .6832643**

**freq | .5465127 .1279315 -2.58 0.010 .345418 .8646802**

**lessfreq | .8567193 .18402 -0.72 0.472 .5623461 1.305189**

**binge2 | .772812 .1549701 -1.29 0.199 .5216564 1.144888**

**abuse | 1.223786 .3408011 0.73 0.468 .7090261 2.112268**

**alcdep2 | 5.221329 1.422967 6.06 0.000 3.060578 8.90756**

**sexprob | 3.743136 .7926717 6.23 0.000 2.471607 5.668809**

**_cons | .1913845 .0310347 -10.20 0.000 .1392761 .2629884**

**-------------------------------------------------------------------------------**
